# Rapid Apta-Chromogenic Detection Method for Nitrofuran Metabolite Determination

**DOI:** 10.3390/molecules29081720

**Published:** 2024-04-10

**Authors:** Navarat Chaisri, Chutikarn Jaengphop, Ikuo Hirono, Sasimanas Unajak

**Affiliations:** 1 Interdisciplinary Graduate Program in Genetic Engineering, The Graduate School, Kasetsart University, Bangkok 10900, Thailand; 2 Department of Biochemistry, Faculty of Science, Kasetsart University, Bangkok 10900, Thailand; chutikarn.ja@ku.th; 3 Laboratory of Genome Science, Graduate School of Tokyo University of Marine Science and Technology, Tokyo 108-8477, Japan; 4 Kasetsart Vaccines and Biologics Innovation Centre, 50 Ngam Wong Wan, Chatuchak, Bangkok 10900, Thailand

**Keywords:** aptamer, nitrofuran metabolites, detection, simultaneous detection, colorimetric detection, apta-chromogenic detection

## Abstract

Nitrofuran (NF) contamination in food products is a global problem resulting in the banned utilization and importation of nitrofuran contaminated products. A novel chromogenic detection method using a specific DNA aptamer with high affinity and specificity to nitrofurans was developed. Single-stranded DNA aptamers specific to nitrofuran metabolites, including 3-amino-2-oxazolidinone (AOZ), 3-amino-5-methylmorpholino-2-oxazolidinone (AMOZ), and 1-aminohydantoin (AHD), were isolated using magnetic bead-SELEX. The colorimetric detection of nitrofurans using gold nanoparticles (AuNPs) exhibited an AOZ detection range of 0.01–0.06 ppb with a limit of detection (LOD) of 0.03 ppb. At the same time, this system could detect AMOZ and AHD at a range of 0.06 ppb and 10 ppb, respectively. The fast nitrofuran extraction method was optimized for food, such as fish tissues and honey, adjusted to be completed within 3–6 h. This novel apta-chromogenic detection method could detect NF metabolites with a sensitivity below the minimum required performance limit (MPRL). This analysis will be valuable for screening, with a shortened time of detection for aquaculture products such as shrimp and fish muscle tissues.

## 1. Introduction

Food safety is an important issue globally, especially for human consumption. Several antibiotics, such as nitrofurans (NFs) and chloramphenicol, have been used in livestock and aquaculture, resulting in the contamination of products by antibiotics and their residuals. Therefore, these agents have been banned in veterinary medicine. NFs are one of the antibiotics previously employed for treating many bacterial and protozoal infections [1,2]. These agents are often used in the treatment of urinary tract infections and vaginal infections as well as dermatitis in animals such as pigs, ducks, chickens, shrimp, and fish [3].

The common structure of NFs is composed of a nitrofuran ring that differs in its side chain attached by an azomethine bond. Due to the short half-life of NFs, NFs in parental forms can be rapidly metabolized to their derivatives, such as 3-amino-2-oxazolidinone (AOZ) for furazolidone, 3-amino-5-methylmorpholino-2-oxazolidinone (AMOZ) for furaltadone, 1-aminohydantoin (AHD) for nitrofurantoin, and semicarbazide (SEM) for nitrofurazone [2,4]. These reactive derivatives can bind covalently to biomolecules in animals, such as DNA and proteins, for several months [4,5], whereas their parental form is scarcely detected in tissues and plasma [4]. Importantly, some forms of NFs, such as 3-amino-2-oxazolidinone (AOZ), can release its side chain, the furazolidone side chain, into ß-hydroxyethylhydrazine, which is recognized as a toxic substance in food products due to its carcinogenic, mutagenic, and teratogenic effects [1,4,6]. However, due to the long-term improper utilization of NFs, the deposition of NFs in the environment could cause ecotoxicological impacts on natural living organisms, such as bacteria, plants, and crustaceans, which compose different levels of the food chain [6]. On the other hand, NF contamination in raw materials and feed ingredients such as fish meal leads to the transfer of residual chemicals to aquatic animals and fisheries products intended for human consumption [7]. Because of this, the prohibition of NF utilization in aquaculture and the limitation of NF residue contamination in aquaculture products are of strict concern in many countries, including countries in the European Union (EU), Japan, and the United States [8], which prohibited their import and mixing in animal feed for treatment in cultivating systems in the EU in 1995 [2].

The monitoring of NFs could only detect tissue-bound metabolites because of the impractical detection of parental drug residues in the edible part [9]. However, as protein-bound metabolites of NFs, AOZ, AMOZ, AHD, and SEM could remain for several months and act as markers for monitoring NF contamination. HPLC–MS/MS is the standard method for monitoring NF residues in animal tissues and other sources due to its reliability in detection and quantification [10]. Several alternative methods for NF detection have been mentioned, such as UPLC–MS/MS [11], ELISA [8,9,12] and the flow injection chemiluminescence (FI-CL) method [13]. However, the limitations of these methods are that they are time consuming, high cost, and labor intensive methods that require sophisticated equipment and skilled personnel to prepare and analyze the samples. In aquaculture systems, a rapid screening method for detecting residual NFs that can be used at cultivation sites or before harvesting would facilitate the prompt detection of residuals before harvesting, freezing, and exporting. This screening method could be used to screen NF contamination in aquatic products, which could reduce expenses and shorten the duration of residual inspection operations.

The application of the nucleotide-based library “aptamer” to mimic antibodies used for recognizing antibiotics and their metabolites, such as aminoglycoside [14], oxytetracycline (OTC) [15], and chloramphenicol [16], was established [17]. These aptamers have been further developed for several types of detection, including colorimetric detection and biosensors [18]. However, recently, no DNA aptamer specific for NFs has been identified. The development of a rapid diagnostic method for NF residuals in fisheries products is an alternative method for screening contaminants prior to harvesting and exporting. Thus, we report the first aptamer specific for NFs and their derivatives and the development of colorimetric detection for AOZ, AMOZ, and AHD in shrimp and fish samples.

## 2. Experimental Methods

### 2.1. Chemicals and Reagents

Furazolidone (3-amino-2-oxazolidinone = AOZ), furaltadone (3-amino-5-morpholinomethyl-2-oxazolidinone = AMOZ), and nitrofurantoin (1-aminohydantoïne = AHD) (Hayashi Pure Chemical, Japan) were dissolved in methanol to obtain a concentration of 1 µg. µL^−1^. Antibiotics, including kanamycin, chloramphenicol, and ampicillin, were purchased from Sigma-Aldrich, St. Louis, MO, USA. Enrofloxacin was purchased from Nutrichems, Thailand, and amoxycillin was purchased from The United Drug, Thailand. Reagents for polymerase chain reaction (PCR) were purchased from Thermo Scientific, USA. Gold nanoparticles approximately 15, 20, and 40 nm in diameter were purchased from Serve Science, Thailand.

### 2.2. Identification of Aptamers Specific to AOZ

The systematic evolution of ligands by exponential enrichment (SELEX) procedure was used to select DNA aptamers specific to AOZ and its derivatives. Briefly, AOZ was conjugated with Dynabeads^®^ M-280 Tosyl-activated magnetic beads (Invitrogen, Waltham, MA, USA) following the instructions, and the efficiency of immobilization was verified using LC–MS/MS (kindly analyzed by Ms. Patcharee Soonsan, Department of Fisheries, Thailand). The aptamer DNA library (5′ TAA TAC GAC TCA CTA TAG GGC CAG GCA GCG AG -N_40_- CC GAC CAC ACG CGT CCG AGA 3′) was incubated with 1 µg.µL^−1^ AOZ at 37 °C for 1 h with rotation to allow aptamer binding with AOZ. Then, the aptamer-bound AOZ-magnetic beads were separated by placement on a magnetic stand and washed three times with sterile PBS. Bound aptamers were then separated from magnetic beads by heating and kept as templates for the next round of selection.

The amplification of bound aptamer was performed by using biotinylated primers (Biotin F: 5′ biotin-TAA TAC GAC TCA CTA TAG GGC CAG GCA GCG AG-3′ and Biotin R: 5′ biotin -TCT CGG ACG CGT GTG GTC GG-3′), and the amplification was performed with 20 cycles with amplification conditions as follows: predenaturation at 95 °C for 3 min, denaturation at 95 °C for 1 min, annealing at 55 °C for 30 s, and extension at 72 °C for 10 s. The PCR product containing biotin was purified using Dynabeads^®^ M-280 streptavidin (Invitrogen, Waltham, MA, USA) and used for binding with stepwise reduction of AOZ concentration until reaching the 10th round. The sequence of the isolated aptamer was determined from ssDNA isolated in rounds 6th–10th. PCR products from each round were ligated to the pGEM T-Easy vector to determine the nucleotide sequence (Macrogen, Seoul, South Korea). Comparative analysis of the identified aptamer was performed by multiple sequence alignment using Clustal Omega [19].

### 2.3. Preparation of Aptamer-Conjugated AuNPs

The AOZ-aptamer was modified and thiolated using PCR with thiol-forward primer (5′-(SH)-(CH_2_)_6_- CTA TAG GGC CAG GCA GCG AG-3′) and reverse primer (5′ R-TCT CGG ACG CGT GTG GTC GG-3′). AOZ-aptamer-conjugated AuNPs were prepared by covalent bonding with S–S bonds. Prior to conjugation, 5′-thiolate DNA was formed separately by heating at 95 °C for 5 min, immediately cooled to 4 °C for 10 min, and kept at room temperature for 5 min before being treated with freshly prepared 50 mM Tris(2-carboxyethyl) phosphine (TCEP, Thermo Scientific, Waltham, MA, USA) to avoid the formation of hairpin structures and undesirable hybridization between the aptamers. Then, aptamer-conjugated AuNPs (Serve Science, Bangkok, Thailand) (ca. 15, 20, and 40 nm in diameter) were deprotected by the addition of 1 µL of 0.5 mM TCEP to a 100 µL aliquot of 250 ng aptamer in the dark for 1 h. Subsequently, 600 µL of the AuNPs was added to 100 µL of pretreated aptamer aliquot reacted in the dark for 24 h under room temperature during which aptamer was self-assembled on the surface of the AuNPs.

### 2.4. Sample Preparation

In this study, two methods of extraction were performed. Frozen fish or shrimp edible tissues were thawed and minced by scissoring, weighed to obtain 1 ± 0.02 g, and fortified with different concentrations of standard AOZ in methanol. The standard method for derivatized NFs was performed according to the FDA protocol with some modification. Briefly, spiked tissues were placed in a 50 mL polypropylene tube, and then, 0.1 M HCl and 1.5 µM 2-nitrobenzaldehyde (2-NBA) were added to the tissues. The sample tubes were incubated at 37 °C overnight in an incubator. On the following day, after allowing the sample to cool to room temperature, 5 mL of 0.1 M K_2_HPO_4_.3H_2_0 and 400 µL of 1 M sodium hydroxide (NaOH) were added and the sample pH was adjusted to 7.0 ± 0.5. Then, 5 mL of ethyl acetate (CH_3_COOC_2_H_5_) was added to each tube and vortexed thoroughly for 1 min. Samples were centrifuged at 2000 rpm for 15 min. Clear lysates of the sample solution were transferred into a glass tube. The extract was then evaporated to dryness at 50 °C in a heat block chamber under a mild flow of nitrogen gas. Dried materials were dissolved in 400 mL of water:methanol solution (1:1) and centrifuged at 8500 rpm for 20 min. Supernatants were then filtered through a 0.2 µm nylon filter.

For the modified extraction method, different parameters were varied, including the concentrations of HCl and NaOH, incubation time, and evaporation method. Briefly, tissue samples were fortified with AOZ. Spiked tissues were placed in a 50 mL polypropylene tube, and then, 0.1 or 0.25 M HCl was added. The mixture was incubated in a 37 °C incubator for 1, 2, 4, 8, 12, and 16 h. Then, the mixture was added to 5 mL of 0.1 M K_2_HPO_4_.3H_2_0 and various concentrations of NaOH from 3–5 M NaOH. The solvent for derivatization was added, and different types of solvents were used, including ethyl acetate, diethyl ether, tertiary butyl methyl ether, acetonitrile, dichloromethane, and chloroform. The extraction followed the common ethyl acetate as described above, except that an air stream was used to dry the extractant. Extractants were used for analysis using LC–MS/MS (Department of Fisheries, Thailand) and aptamer colorimetric detection.

### 2.5. Colorimetric Detection of Samples Using Aptamer-Conjugated AuNPs

Five microliters of standard AOZ with different concentrations (0.01, 0.02, 0.03, 0.04, 0.05, 0.06, 0.07, 0.08, 0.09, 0.1, 0.5, 1, 5, and 10 ppb in methanol) or extracted samples were added to 42.5 µL of aptamer-conjugated AuNP suspension. After addition of an appropriate concentration of NaCl (0, 0.02, 0.04, 0.06, 0.08, 0.1, 0.12, 0.14, 0.16, 0.18, and 0.2 M), the mixture was mixed thoroughly and stood for 2 min at room temperature. The absorbance of the compounds was measured at 630 and 520 nm. The limit of detection (LOD) was determined.

### 2.6. Specificity of Aptamer-Conjugated AuNPs and Analysis of AOZ in Spiked Samples

To determine the specificity of aptamers to NF metabolites but not with other antibiotics, different antibiotics were used to determine the specificity of the system, including 10 ppm kanamycin, ampicillin, enrofloxacin, amoxycillin, and chloramphenicol. Alternatively, fish and shrimp edible tissue were taken from Nile tilapia (*Oreochromis niloticus*) and Pacific white shrimp (*Penaeus vannamei*) carcasses, and honey from a local store was used for spiking with AOZ. A known amount of AOZ (0.1 ppb final concentration) was added to the samples and then allowed to stand for 15 min before the extraction process.

## 3. Results and Discussion

NFs are listed as unapproved drugs in food-producing animals such as livestock and aquatic animals and are not allowed to contaminate food such as honey due to human health concerns. However, NFs (furazolidone, furaltadone, nitrofurantoin, and nitrofurazone) are rapidly transformed into tissue-bound metabolites, and the determination of parental drugs is difficult or impossible to elucidate. Therefore, their metabolites are marked as marker contaminant residues, including AOZ (3-amino-2-oxazolidinone), AMOZ (3-amino-5-morpholinomethyl-2-oxazolidinone), AHD (1-aminohydantoin), and SEM (semicarbazide).

Several studies have been conducted to find the best methodology to identify these residues. Most of the methods require (1) sophisticated instruments, such as liquid chromatography–mass spectrometry (LC–MS), gas chromatography–mass spectrometry (GC–MS), and high-performance liquid chromatography (HPLC), (2) skilled scientists, and (3) additional costs and time for evaluation. However, if a sensitive and reliable screening method is available, it will reduce the risk of residual chemical contamination in food products. Mainly, an immune-based assay.

### 3.1. Screening and Identification of ssDNA Aptamers Specific to AOZ

**Figure 1:**
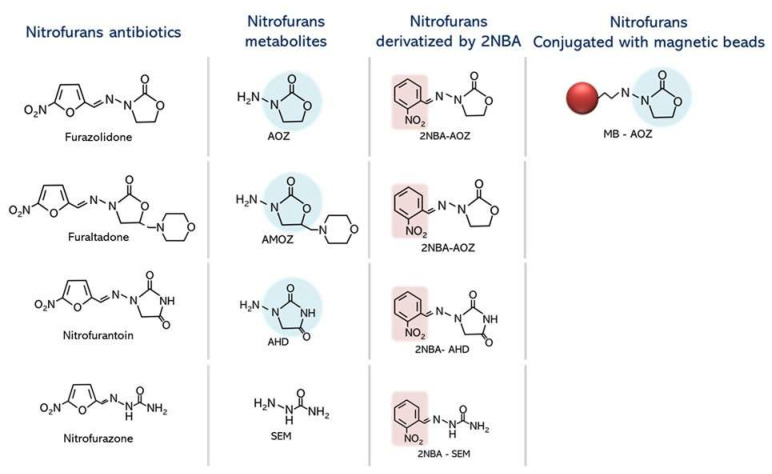
Chemical structures of nitrofurans and their respective metabolites. Derivatives of the tissue-bound residues of nitrofuran antibiotics from acid hydrolysis and 2NBA derivatives were demonstrated. AOZ, a nitrofuran metabolite, conjugated magnetic beads were demonstrated. The light blue circle represents a similar structure of NF metabolites recognized by the AOZ aptamer.

Due to the small size of NFs, the extension of the NF structure was assisted by tosyl-activated magnetic beads to allow the NFs to bind with the ssDNA aptamer library. Regarding the similar structures of the NF metabolites furazolidone (AOZ), furaltadone (AMOZ), and nitrofurantoin (AHD) (Figure 1), AOZ was used as a candidate NF metabolite. Prior to screening with the ssDNA aptamer, AOZ-conjugated magnetic beads were evaluated for their capacity to load AOZ using LC–MS/MS, which indicated 67% NF conjugation. Through SELEX analysis, the nucleotide sequence of ssDNA aptamers from the 6th–10th rounds was determined. Five different ssDNA aptamer sequences were isolated (Supplemental Data S1).

### 3.2. Colorimetric Aptamer–AuNP-Based Colorimetric Detection

Regarding the visual change in the color of gold nanoparticles (AuNPs), five different ssDNA aptamers were synthesized and modified with thiol groups that allowed aptamer immobilization on the AuNP surface. In the presence of AOZ, the color of the AuNP solution changed from red to blue/purple, which allowed for easy interpretation and could be chosen for suitable aptamers.

Among the five different isolated aptamers, only the aptamer with the highest redundant sequence exhibited a change in the presence of AOZ (Supplementary Data S1). Hence, this specific aptamer was isolated from the 7th–10th rounds of SELEX screening. This evidence affirmed that the enrichment of the ssDNA aptamer specific to AOZ was increased in relation to the increase in the round of SELEX, while most of the nonspecific or low affinity ssDNA aptamer was eliminated after a reduction in AOZ concentrations. Moreover, screening this specific aptamer to small molecules was achieved by extending their structure to be exposed to the ssDNA aptamer library with magnetic beads. Currently, several specific aptamers against antibiotics have been isolated, such as aptamers specific to β-lactams, aminoglycosides, anthracyclines [18], chloramphenicol, (fluoro)quinolones, lincosamide, tetracyclines, and sulfonamides.

### 3.3. Optimization of Colorimetric Aptamer–AuNP-Based Colorimetric Detection

**Figure 2:**
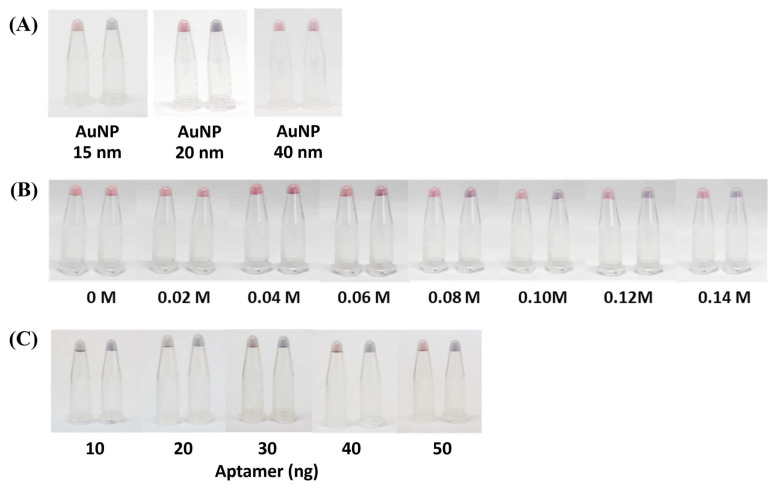
The effect of AuNP size and NaCl concentration on aptamer–AuNP color alteration. (**A**) AuNPs sized 15, 20, and 40 nm clearly altered the aptamer–AuNP color change from red to purple-blue in the presence of AOZ. (**B**) NaCl concentration (0–0.14 M) for the color change of the aptamer—AuNP–AOZ colloid complex. (**C**) Aptamer concentration (10, 20, 30, 40, 50 ng) was optimized for color development conditions by varying the concentration.

To optimize and generate distinct coloration of the AuNP complex, the AuNP size (15, 20, and 40 nm), the concentration of the NaCl (0.00–0.20 M,), and the concentration of the aptamer (10–50 ng) were adjusted in this AOZ aptamer–AuNP-based colorimetric detection method. With the equivalent sizes of AuNPs, the concentration of the NaCl and aptamer can influence the coloration, as the AuNPs (20 nm) could make clear and distinct purple colors in the presence of AOZ, while 15 and 40 nm AuNPs showed similar colors to those of the negative control (Figure 2A). Alternatively, a concentration of NaCl greater than 0.06 M could generate a color change in the AuNP–aptamer in the presence of AOZ (Figure 2B). The aptamer concentration at 50 ng showed the appropriate salt-induced aggregation with a clear color change (Figure 2C).

**Figure 3:**
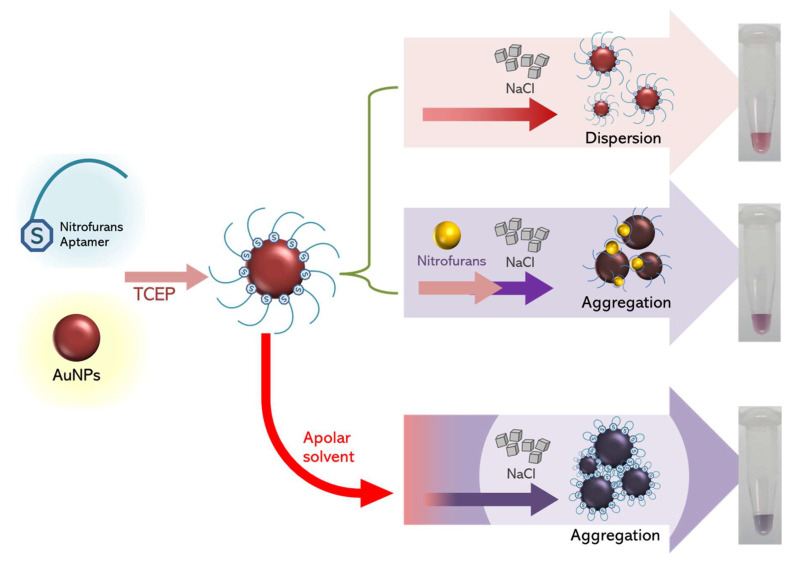
Principle of colorimetric determination of AOZ based on aggregation of modified gold nanoparticles (AuNPs).

It was previously mentioned that larger AuNPs should elicit a greater response of AuNP aggregation, but in this study, the largest AuNPs (40 nm) did not agree with the previous conclusion [20]. To explain the effect of AuNP size on color development, we hypothesized that the size of AuNPs might reflect the surface available to be adsorbed by the aptamer. As the aptamers were adsorbed on the surface of AuNPs, when AOZ molecules were present, the aptamer could interact with AOZ, change the AuNP surface properties, and allow AuNP aggregation (Figure 3). Then, the AOZ concentration increased, resulting in a shift in AuNP aggregation [21]. Thus, the ratio of AuNPs and aptamer in the largest AuNPs (40 nm) was not favorable for binding with the target and affected the performance of AuNP-aptamer-AOZ aggregation [21].

The optimization of NaCl concentration is a promising step which allows for the stability of AuNPs-based colorimetric assay. With a lower concentration of NaCl, AuNP aggregation could not be induced and the color of the AuNP solution did not change in the presence of NFs. On the other hand, too high a NaCl concentration could destroy the stability of the AuNP–aptamer and the color of the AuNP solution could change immediately before adding the NFs (Figure 2, Supplementary Data S2) [21]. Thus, in this study, with a specific concentration of the AuNPs–aptamer, the optimization of the NaCl concentration was seen as the clear purple color solution with 0.10 M NaCl. However, based on the high sensitivity of detection, the aptamer (50 ng) conjugating AuNPs (20 nm) with 0.10 M NaCl was considered a suitable condition for further analysis.

### 3.4. Sensitivity and Specificity of AOZ Detection

**Figure 4:**
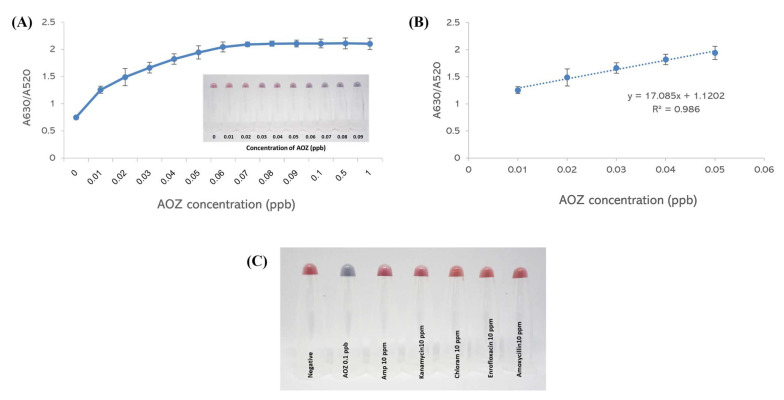
Sensitivity and specificity of aptamer–AuNP-based colorimetric detection. (**A**) Absorbance characteristics of AuNPs and visual observation of AuNPs with different concentrations of AOZ. (**B**) The linear range of AOZ determination using aptamer–AuNP-based colorimetric detection. (**C**) Visual observation of AuNPs with different types of antibiotics.

To evaluate the sensitivity of the AOZ detection method, the A630/A520 absorption ratio was monitored and showed an increase in absorbance with increasing AOZ concentration. The color of the aptamer–AuNPs changed from red to purple (inset of Figure 4A) as the AOZ concentration increased. A steep increase in A630/A520 corresponded to an increase in AOZ concentration from 0.01–0.06 ppb, and a shallow increase was detected afterward (Figure 4B). This evidence demonstrates that the saturation of AuNP aggregation might be achieved with 0.07 ppb and above. The detection limit (LOD) of the system was calculated to be 0.03 ppb. Moreover, this aptamer was specific to other nitrofuran metabolites, including 3-amino-5-morpholinomethyl-1,3-oxazolidin-2-one (AMOZ) and 1-amino-hydantoin (AHD), with limits of detection of 0.06 ppb and 10 ppb, respectively (Supplementary Data S3). Compared to the other existing techniques for determining nitrofurans, such as UPLC-DAD, HPLC-DAD, LC–MS/MS, and ic-ELISA, this colorimetric detection assay exhibits a greater sensitivity with a lower LOD, as summarized in Supplementary Data S4.

To evaluate the specificity of the AOZ detection method, other antibiotics used in animal production, including 10 ppm kanamycin, ampicillin, enrofloxacin, amoxycillin, and chloramphenicol, were tested. None of these antibiotics exhibited significant color changes compared with AOZ (Figure 4C). These results demonstrated that this aptamer–AuNP colorimetric method is highly specific to AOZ.

### 3.5. Optimization of the AOZ Extraction Method

A common method for extracting AOZ residues from fish and shrimp tissues for LC–MS/MS analysis was used in this colorimetric detection method. However, a disadvantage of this common extraction method is that it is time-consuming, taking longer than 18 h for extraction, and uses special equipment for extraction, such as nitrogen gas for evaporation. Thus, the optimization of the NF extraction process was developed to shorten the extraction time and use simple equipment. Four factors, including the type of solvents, the concentration of HCl, the concentration of NaOH, and the evaporation process were considered for optimization.

#### 3.5.1. Type of Solvent

**Figure 5:**
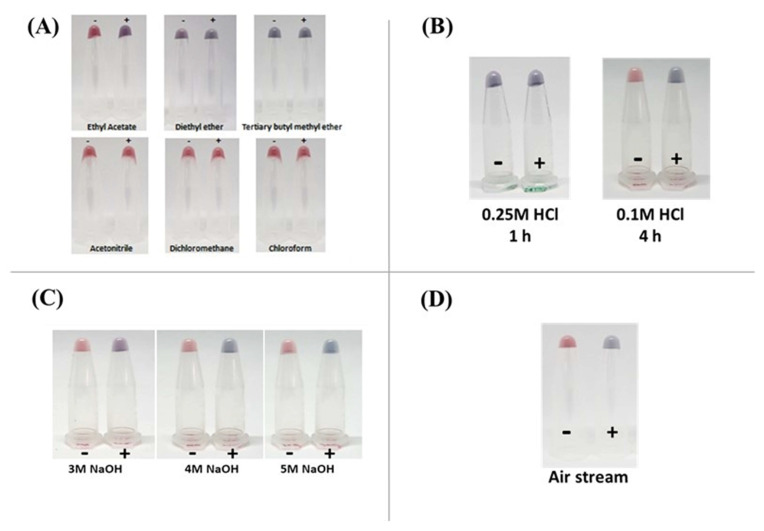
Optimization of the AOZ extraction method. (**A**) Effect of solvents used for extraction. Six types of solvents were chosen and used in the extraction of spiked samples, including ethyl acetate, acetonitrile, dichloromethane, tertiary butyl methyl ether, diethyl ether, and chloroform. (**B**) Effect of HCl concentration. Fish tissues were extracted for NFs using common methods. (Left) 0.25 M HCl was added instead of 0.25 M HCl and incubated for 1 h, (Right) 0.1 M HCl was used and incubated for 4 h. (**C**) Effect of NaOH concentration. Fish tissues were extracted by using 0.1 M HCl and incubated for 4 h at various concentrations (3, 4, 5 M NaOH). (**D**) Effect of the drying method. After extraction, the extractants were dried with an air stream, and the dried materials were dissolved and tested with an aptamer-AuNP detection assay. (-): extractant from fish tissue without AOZ. (+): extractant from fish tissue spiked with 1 ppb AOZ.

Ethyl acetate, a moderately polar organic solvent, was used to extract nitrophenyl derivatives from tissues [2]. In this study, five alternating solvents, including diethyl ether, tertiary butyl methyl ether, acetonitrile, dichloromethane, and chloroform, were used to extract AOZ from fish tissues (Figure 5A). The extractant was subjected to analysis on the AuNP–aptamer system, revealing that only the extractant from ethyl acetate could extract AOZ without interfering with the AOZ aptamer–AuNP colorimetric detection system. However, diethyl ether and tertiary butyl methyl ether exhibited a significant change in the color of the AuNP–aptamer system to purple coloration without the addition of target analytes (Figure 5A). It should be noted that on the surface of the AuNPs, the structure of DNA aptamers was immediately changed in a polar solvent and changed their solubilization properties, in which this DNA aptamer was no longer able to dissolve in this polar solvent [22]. However, other solvents, including acetonitrile, dichloromethane, and chloroform, could not extract AOZ and showed red coloration in the presence of AOZ (Figure 5A). Therefore, it is necessary to use ethyl acetate in the extraction process.

#### 3.5.2. Concentration of HCl

In the standard NF extraction method, 16 h are required to extract NF from animal tissues. Two major extraction and derivatization steps include releasing NF from the tissues by using mildly acidic conditions and NF derivatization [2]. In this study, to fasten the NFs for extraction, the evaluation of NF extraction took place by varying several parameters, including incubation time, acid concentration, and incubation temperature.

First was the reduction of incubation time to 1 h and treatment with 0.1 M HCl. None of the NF could be extracted from animal tissue as determined using colorimetric detection. This could be explained as the reduction of incubation time would directly affect the release of NF through the lack of the success of metabolite–protein bond hydrolysis. Second, the concentration of HCl was increased to 0.25 M, and the incubation temperature varied between37 °C, 50 °C, and 65 °C. However, the failure to extract NF was observed upon increasing the concentration of HCl and varying the incubation temperature (Figure 5B). Therefore, the original 0.1 M HCl acid extraction step continues to be used for NF extraction.

#### 3.5.3. Concentration of NaOH

After acid hydrolysis and derivatization, the pH of the extractant must be adjusted by NaOH. To shorten the extraction time, the concentration of NaOH varied between 3, 4, and 5 M. Successful of pH adjustment was observed using 3, 4, and 5 M NaOH for 4 h (Figure 5C). Therefore, to ensure pH neutralization, we used 4 M NaOH to adjust the pH of the extractant.

#### 3.5.4. Evaporation Process

Nitrogen gas is normally used to remove the organic solvents from the extractant in the standard NFs extraction method. However, a limitation in the use of nitrogen gas and its additional equipment is the additional cost, and the gas might not be supplied in some local areas. Therefore, an alternative extractant drying method was considered. In this study, an aquarium air pump replaced the nitrogen gas. This had the same result as nitrogen gas evaporation, that is, the color changed from the red to blue in the presence of AOZ (Figure 5D). This suggested that the air streaming method could replace the nitrogen gas in drying the extractant. Moreover, it should also be noted that the use of an aquarium air pump to provide an air stream in this drying method can be used for on-site NF extraction, especially in fish and shrimp cultivation areas.

In addition, the derivatization of NF using 2-NBA is required in all reported procedures—including the standard protocol. These derivatized NFs could be used in different detection methods such as HPLC/MS [7], biochip screening assays [23], automated SPE, and LC–MS/MS [24]. Even though 2-NBA is commonly used to increase the molecular mass of NF [2], this reagent interfered with AuNP colorimetric detection by changing the color of the naked AuNP–aptamer to deep purple. This circumstance was consistent with contamination with 2-NBA or the excessive use of solvents such as hexane to remove 2-NBA contaminants reflected in high-performance liquid chromatography-diode array detection (HPLC-DAD) [25]. Successful AOZ detection without 2-NBA derivatization could be explained by the specificity of the aptamer to the original structure of AOZ and its derivatives (Figure 1). On the other hand, the screening of AOZ was performed in an aqueous system. Therefore, contamination with an organic solvent such as hexane might interfere with the aptamer’s structure and affect binding between aptamer and AOZ. This circumstance resembled the preparation of NF for MS analysis and immunoassay which does not require the modification or derivatization of their structure [26].

To obtain a rapid AOZ screening analysis, a reduction in the process and time needed for NF extraction from biological tissues was considered. Most of the standard NF extraction protocols for MS/MS analysis and ELISA require at least 16 h. In this study, the extraction time was reduced from 16 h to 5 h by alternating (1) the incubation period for solvent extraction, (2) the dry evaporation process, (3) the concentration of HCl and NaOH, and (4) the incubation temperature. NF extraction for aptamer detection was completed using 0.1 M HCl with ethyl acetate solvents and incubated with 4 M NaOH for 5 h at room temperature. This NF extraction protocol was similar to the optimized sample extraction protocol for HPLC-DAD detection [25].,except the air stream replaced the nitrogen gas drying process. However, the preparation time was within 6 h, which acceptable for the extraction of contaminants from animal tissues [24]. Taken together, it could be summarized that this modified NF extraction method for apta-chromogenic detection is faster than previous NF extraction methods [7,23,27].

### 3.6. Validation on Other Samples and Honey

**Figure 6:**
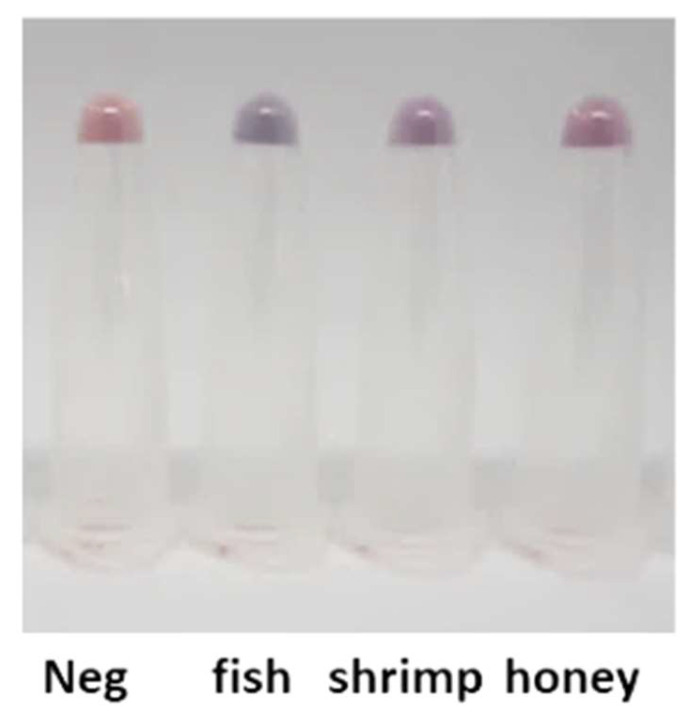
Another nitrofuran extraction sample. Fish tissues, shrimp tissues, and honey spiked and not spiked with NFs were incubated with 1 M HCl for 4 h to extract the residual chemical. The extractant was determined for NFs using aptamer–AuNPs.

Using the developed AOZ AuNP–aptamer system and optimized extraction protocol, the NF-contaminated fish tissues, shrimp tissues, and honey (Doikham Natural Honey, Thailand and Coles 100% Australian Pure Honey Squeeze, Australia) were validated. All samples were spiked with 0.04 ppb AOZ. All samples were extracted by incubating with 1 M HCl for 4 h to extract the residual chemical. The results indicated that the system could detect AOZ contamination in all tested samples (Figure 6). In addition, the validation of this AuNP–aptamer system on AMOZ and AHD was achieved, but demonstrated different detection sensitivities for AMOZ (0.06 ppb) and AHD (100 ppb) (Supplementary Data S1). However, it should be noted that another nitrofuran, SEM, could not be detected by this system. Therefore, the development of an aptamer specific to SEM will be further elucidated.

The minimum required performance limit (MRPL) for nitrofuran metabolites in poultry and aquaculture products allows NF contamination to be set at 1.0 mg/kg or 1 ppb [28]. Our apta-chromogenic detection can measure the combination of AOZ at a limit of detection (LOD) of 0.03 ppb (0.03 µg/kg), which is compatible with the requirement of authorized food-producing animals in the EU. The sensitivity of the assay is greater than the quantitative analysis of nitrofuran metabolites in shrimp using the liquid chromatograph-tandem mass spectrometric detection analytical range method for NFs of 0.25–5.0 µg/kg [29]. Alternatively, the quantitation of NF metabolites in aquaculture products using microwave-assisted derivatization, automated SPE, and LC–MS/MS has detection limits of ≤0.06 ng/g, with a quantitation limit of ≤0.2 ng/g [24]. Moreover, the comparison with immunodetection using a specific antibody revealed that our detection module exhibits greater sensitivity of detection than other methods, such as the lateral flow biosensor for multiplex detection of nitrofuran metabolites capable of visually detecting four metabolites at the same time with a detection limit of 0.1 ppb (1 μg/L) [26]. Moreover, the immunochromatographic test showed four nitrofuran metabolites in fish samples: 0.5 ppb (0.5 ng/mL) for AOZ and 0.75 ppb (0.75 ng/mL) for AHD, SEM, and AMOZ [30]. The detection of antibiotics in honey using a multispot nanoarray detected nitrofuran (AMOZ, AOZ, SEM, AHD) and chloramphenicol with detection limits of 0.23, 0.98, 24.8, 58.9 and 0.24 ppb (ng/mL), respectively [31]. On the other hand, it is known that the colorimetric method using AuNPs provides a rapid detection time within 5 min [32,33,34], which is similar to our system. Taken together, this research demonstrated the simultaneous and sensitive detection of NF metabolites in aquaculture products.

## 4. Conclusions

A novel apta-chromogenic detection method was developed to detect NFs. The extraction method was shortened to 5 h and omitted the 2-NBA modification. The detection time was shortened to less than 15 min. This system could be used for the rapid and simultaneous screening of contaminating agents in biological sources and could be used as an alternative method in screening for NF contamination. Importantly, the sensitivity of AOZ reached 0.03 ppb, which approached the limited extent of MRP and could be used to detect AOZ contamination in aquatic animals and honey.

## Data Availability

Data will be made available on request.

## Supplementary Materials

The following supporting information can be downloaded at https://www.mdpi.com/article/10.3390/molecules29081720/s1. Supplemental Data S1: Nucleotide sequence of ssDNA aptamer to AOZ. Colorimetric detection of AuNPs—aptamer to AOZ was demonstrated. The number of identical nucleotide sequences with each round of SELEX screening is shown. Supplemental Data S2: Colorimetric determination of AuNPs solution: (1) solution of AuNP with NaCl; (2) solution of AuNP—aptamer complex in the presence of NaCl; and (3) solution of AuNP—aptamer complex in the presence of NaCl and AOZ. Supplemental Data S3: Single-stranded DNA aptamer specific to nitrofuran metabolites, including (A) 3-amino-5-morpholinomethyl-1,3-oxazolidin-2-one (AMOZ) and (B) 1-amino-hydantoin (AHD), with limits of detection of 0.06 ppb and 10 ppb, respectively. Supplemental Data S4: Comparison of different techniques for the detection of nitrofurans or AOZ in aquatic animal products.
