# Supplementary material for: Rapid Apta-Chromogenic Detection Method for Nitrofuran Metabolite Determination
Source: Molecules. 2024 Apr 10;29(8):1720. doi: 10.3390/molecules29081720 (PMC11052085; doi:10.3390/molecules29081720)
Supplement: Supplementary file 1 [file molecules-29-01720-s001.zip › molecules-2526419-supplementary.pdf]

Supplement data

Rapid Apta-Chromogenic Detection Method for Nitrofuran Metabolite Determination

Navarat Chaisri, Chutikarn Jaengphop, Ikuo Hirono, Sasimanas Unajak

SUPPLEMENT Data

**Supplemental Data S1**	Nucleotide sequence of ssDNA aptamer to AOZ. Colorimetric detection of AuNPs – aptamer to AOZ was demonstrated. The number of identical nucleotide sequences with each round of SELEX screening is shown.
**Supplemental Data S2**	Colorimetric determination of AuNPs solution: 1) solution of AuNP with NaCl; 2) solution of AuNP – aptamer complex in the presence of NaCl; and 3) solution of AuNP - aptamer complex in the presence of NaCl and AOZ.
**Supplemental Data S3**	Single-stranded DNA aptamer specific to nitrofuran metabolites, including (A) 3-amino-5-morpholinomethyl-1,3-oxazolidin-2-one (AMOZ) and (B) 1-amino-hydantoin (AHD), with limits of detection of 0.06 ppb and 10 ppb, respectively.
**Supplemental Data S4**	Comparison of different techniques for the detection of nitrofurans or AOZ in aquatic animal products.

Supplement Data S1

**Number of identical sequences**	**AuNPs colorimetric test**	**Aptamer clone**
9	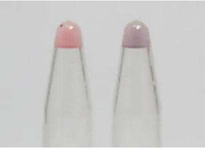	7th round: 3 clones<br>9th round: 2 clones<br>10th round: 4 clones
2	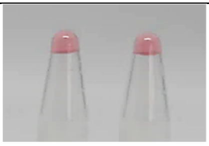	6th round: 2 clones
2	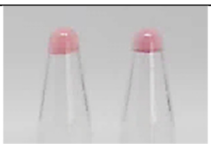	6th round: 2 clones
2	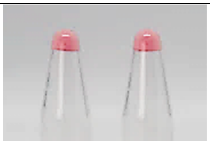	7th round: 2 clones
2	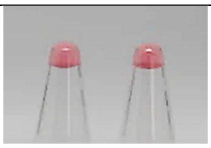	6th round: 2 clones

Supplement Data S2

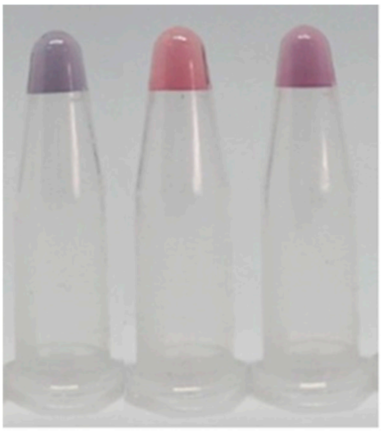

1 2 3

1 = AuNP + NaCl
2 = AuNP-Apt + NaCl
3 = AuNP-Apt + AOZ + NaCl

Supplement Data S3

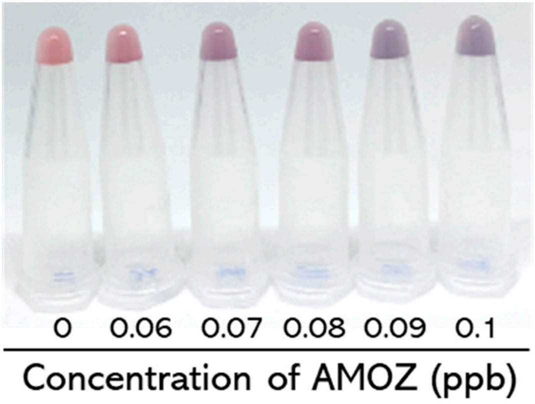

(A)

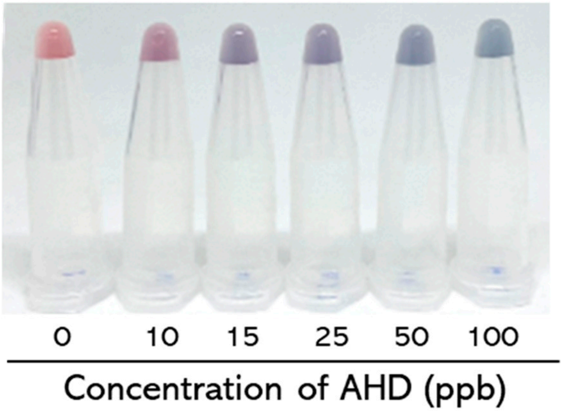

(B)

Supplement Data S4

No.	Name of the antibiotic	Type of technique	Biorecognition element	LOD	Reference
1	Nitrofuran	Liquid Chromatography-Photodiode Array Detection (UPLC-DAD)	2-nitrobenzaldehyde (2-NBA)	0.25−0.33 µg/kg	Wang *et al*., 2003
2	Nitrofuran	High-performance liquid chromatography with a diode-array detector (HPLC-DAD) and liquid chromatography-tandem mass spectrometry (LC–MS/MS)		0.2–0.4 µg/L	Yu *et al*., 2013
3	3-amino-2-oxazolidinone (AOZ)	Enzyme-linked immunosorbent assay (ic-ELISA)	2-nitrobenzaldehyde (2-NBA)	0.15 and 0.3µg/kg	Liu *et al*., 2010
4	Nitrofuran	High Performance Liquid Chromatography Diode Array Detection (HPLC-DAD)		1 µg/kg	Fernando *et al*., 2017
5	3-amino-2-oxazolidinone (AOZ)	Apta-Chromogenic Detection		0.03 µg/kg (ppb)	This work
